# Which Came First? When Usher Syndrome Type 1 Couples with Neuropsychiatric Disorders

**DOI:** 10.3390/audiolres13060086

**Published:** 2023-12-11

**Authors:** Paola Tesolin, Aurora Santin, Anna Morgan, Stefania Lenarduzzi, Elisa Rubinato, Giorgia Girotto, Beatrice Spedicati

**Affiliations:** 1 Department of Medicine, Surgery and Health Sciences, University of Trieste, 34149 Trieste, Italy; paola.tesolin@burlo.trieste.it (P.T.); giorgia.girotto@burlo.trieste.it (G.G.); beatrice.spedicati@burlo.trieste.it (B.S.); 2 Institute for Maternal and Child Health—I.R.C.C.S. “Burlo Garofolo”, 34137 Trieste, Italystefania.lenarduzzi@burlo.trieste.it (S.L.); elisa.rubinato@burlo.trieste.it (E.R.)

**Keywords:** Usher syndrome type I, *CDH23*, psychiatric symptoms

## Abstract

Usher syndrome (USH) is an autosomal recessive disorder characterized by sensorineural hearing loss (HL), retinopathy, and vestibular areflexia, with variable severity. Although a high prevalence of behavioural and mental disorders in USH patients has been reported, few studies on these psychiatric and psychological issues have been conducted. This work describes the case of a 16-year-old boy affected by congenital bilateral sensorineural HL, presenting a suddenly altered behaviour concomitant with a decrease in visual acuity. To establish a molecular diagnosis, Whole-Exome Sequencing analysis was performed, detecting a pathogenetic homozygous variant (c. 5985C>A, p.(Tyr1995*)) within the *CDH23* gene. *CDH23* is a known USH type 1 causative gene, recently associated with schizophrenia-like symptoms and bipolar disorders. To date, no studies have provided evidence of a direct genotype–phenotype correlation between USH patients carrying *CDH23* variants and mental/behavioural issues; however, considering the multiple biological functions of *CDH23*, it can be hypothesised that it could have a pleiotropic effect. Overall, this study highlights the relevance of a continuous clinical evaluation of USH patients, to monitor not only the disease progression, but to early detect any psychological or behavioural alterations, thus allowing a rapid implementation of therapeutic strategies aimed at improving their quality of life and well-being.

## 1. Introduction

The majority of patients concomitantly affected by genetic deafness and blindness are diagnosed with Usher syndrome (USH), which affects 1 per 10,000 Northern European and American individuals [1,2].

USH is an autosomal recessive disorder characterized by sensorineural hearing loss (HL), retinitis pigmentosa (RP), and in some cases, vestibular areflexia [3]. Additionally, this syndrome presents with a clinical and genetic heterogeneity, and it is classified in three main different clinical subtypes. Patients affected by USH type 1 (USH1) display profound congenital deafness, vestibular dysfunction, and prepubertal RP. To date, USH1 has been associated with pathogenic variants within five different genes, namely *USH1C*, *MYO7A*, *CDH23*, *PCDH15*, and *USH1G* [2,4]. Mild-severe congenital HL characterizes USH type 2 (USH2), while RP appears in adolescence. USH2 may be caused by mutations within *USH2A*, *ADGRV1*, and *WHRN* [2,4]. Finally, USH type 3 (USH3) is associated with rapidly progressing HL with variable age of onset for the vestibular phenotype and RP, and it is caused by variants within the *CLRN1* gene [2,4].

A thorough search of the literature highlights an association between USH and various mental disorders, mainly schizophrenia-like psychosis [1,4]. In particular, some studies, mostly case reports and a few observational dated studies, described a co-occurrence of USH and persecutory delusions, depression, mental and behavioural issues, anorexia, panic attacks and anxiety, paranoid schizophrenia, or psychotic disorders [5,6,7,8,9].

Despite these reports, to date, there is still fragmented information regarding the relationship between USH and psychiatric issues, and there are no defined genotype–phenotype correlations. Furthermore, the exact definition of their prevalence still needs clarification, with estimations ranging between 4% and 23% [1]. Indeed, in some cases, the definition of a diagnosis may be hampered by the complex interaction with a patient affected by a severe sensory impairment [10]. As a result, a definitive explanation for the enrichment of mental disorders in USH patients has not been highlighted.

Here, we provide a clinical and genetic description of a patient affected by USH1 and displaying psychiatric symptoms.

## 2. Materials and Methods

The proband and his family were recruited by the Medical Genetics Unit of the Institute for Maternal and Child Health—I.R.C.C.S. “Burlo Garofolo” (Trieste, Italy).

The proband underwent a detailed clinical examination, which included audiometric testing and an ophthalmological assessment comprising fundus oculi evaluation, a visual field test, and an electroretinography.

Genomic DNA was extracted from peripheral whole-blood samples of the patient and his parents using the QIAsymphony^®^ SP instrument with QIAsymphony^®^ Certal Kits (Qiagen, Venlo, The Netherlands), following the manufacturer’s instructions. DNA concentration and quality were defined with the Nanodrop ND 1000 spectrophotometer (NanoDrop Technologies Inc., Wilmington, DE, USA).

SNP-arrays were performed with the Infinium Global Screening Array-24 v3.0 BeadChip (Illumina Inc., San Diego, CA, USA), containing 654,027 markers. Illumina’s Genome Studio software v2.0.3 (cnvpartition 3.2.0) was employed to normalize raw image intensity data, genotype clustering, and individual sample genotype calls.

Whole-Exome Sequencing (WES) was carried out on an Illumina NextSeq 550 instrument (Illumina Inc., San Diego, CA, USA). Genomic libraries were prepared with the Twist Human Core Exome + Human RefSeq Panel kit (Twist Bioscience, South San Francisco, CA, USA), according to the manufacturer’s protocol. FASTQ files were processed through a custom pipeline (Germline-Pipeline) developed by enGenome srl (https://www.engenome.com/, Pavia, Italy, accessed on 1 September 2023). Germiline-Pipeline consists of several steps, including FASTQ trimming, FASTQ quality check, FASTQ mapping, marking of duplicates, base quality score recalibration, and variant calling, thus permitting the identification of single-nucleotide variants (SNVs), short insertions/deletions (INDELs), and exon-level Copy Number Variants (CNVs) starting from sequence reads, and finally leading to the generation of VCF files. VCF files were analysed using enGenome Expert Variant Interpreter (eVai) software (https://www.evai.engenome.com/, accessed on 13 November 2023). For the tertiary analysis, the following criteria were applied:

- Variant frequency—Minor allele frequency cut off: 0.1%;
- Variant effect: coding and splicing variants;
- Pathogenicity: ACMG/AMP criteria;
- Family inheritance: autosomal recessive, X-linked recessive;
- Quality of the variant call: ≥20.

The frequency of variants was verified in the Genome Aggregation Database (gnomAD) (https://gnomad.broadinstitute.org/, accessed on 13 November 2023), which contains a description for all the variants identified in the general and healthy population. The pathogenicity of variants was assessed through the Human Gene Mutation Database^®^ (HGMD^®^) (https://my.qiagendigitalinsights.com/bbp/view/hgmd/pro/start.php, accessed on 13 November 2023 [11]) and Deafness Variation Database (http://deafnessvariationdatabase.org/, accessed on 13 November 2023 [12]), which are databases that include possible disease-associated variants described in the literature. The variants’ effects were evaluated using in silico prediction tools such as PolyPhen-2 [13], Sorting Intolerant From Tolerant (SIFT) [14], Pseudo Amino Acid Protein Intolerance Variant Predictor (PaPI score) [15], and Deep Neural Network Variant Predictor (DANN score) [16]. Moreover, the variants were discussed within a multidisciplinary team, and finally confirmed by Sanger sequencing in the proband and his parents.

## 3. Results

The proband is a 16-year-old Caucasian boy born to healthy parents (II:2, II:3, Figure 1). The pregnancy was spontaneous and ended prematurely at 30 weeks and 5 days of gestation. No consanguinity is reported between the parents, but both were born in a small village in north-east Italy with less than 1000 inhabitants. The proband’s younger brother (III:3, Figure 1) is reported to be in good health. The family history of the proband is negative for congenital sensory defects, malformations, and intellectual disability.

In detail, the proband was diagnosed with profound bilateral HL at birth. In particular, the newborn hearing screening detected a bilateral hearing loss, subsequently confirmed with the Auditory Brainstem Response test.

The proband received a first cochlear implant at two years old, and the second at seven years of age. Computed tomography was performed when he was seven years old, revealing a normal inner ear morphology. Other than a slightly impaired equilibrium, no additional symptoms were described during early childhood. In elementary school, the proband received additional support from a dedicated teacher, but no cognitive impairment was detected. First-tier genetic testing, including *GJB2*, *GJB6*, and *SLC26A4* analyses, did not detect any pathogenic variants possibly associated with his phenotype. No additional clinical and genetic examinations were carried out until the proband was 16 years old.

At this age, the proband came to medical attention due to the sudden worsening of his visual acuity. Therefore, the patient underwent electroretinography, revealing highly impaired retinal functionality, with both scotopic and photopic characteristics.

Furthermore, during the clinical examination, the patient displayed an avoidant behaviour. His parents reported that he recently developed insomnia and aggressive behaviours, concomitant with high school beginning and the onset of visual impairment. Notably, the proband’s behavioural problems became so severe that they started to strongly interfere with his everyday life, to the point that he began to refuse to attend school lessons.

Therefore, the proband underwent several neuropsychiatric evaluations with a psychologist, a neuropsychiatrist, and a psychotherapist. Thus, he started treatment with 20 mg/day of fluoxetine. Unfortunately, the patient began to display hetero-aggressive behaviour, so the pharmacological treatment was then combined with 0.5 mg/day of risperidone, subsequently increased to 1 mg/day.

Considering the recent development of additional symptoms, further genetic analyses, as SNP-arrays and WES, were performed. SNP-arrays analysis excluded the presence of micro-deletions/duplications. In particular, no significant CNVs were detected in the proband and his parents. However, three large homozygosity regions were identified in the proband. Specifically, the first one of 15 Mb was detected in 4q32.2.q34.2, while the other two of 10 Mb and 6 Mb were identified, respectively, in 10q21.3q22.2 and 13q32.2q33.1.

Concerning WES data, analysis results revealed the presence of a homozygous variant, c.5985C>A, p.(Tyr1995*) within the *CDH23* (NM_022124.6) gene, inherited from both parents. Notably, the *CDH23* gene is located in the 10q22.1 genomic region, which is within one of the three large homozygosity regions identified by the SNP-arrays analysis.

The variant is not described in the gnomAD (https://gnomad.broadinstitute.org/, accessed on 13 November 2023). It is predicted to be damaging by the in silico prediction tools employed during the analysis (e.g., PaPI, DANN score), and as pathogenetic according to the commonly adopted ACMG/AMP guidelines [17]. Indeed, considering that nonsense variants within *CDH23* are a known cause of USH, it was possible to apply the criteria *Pathogenic Very Strong 1* (PVS1) [17]. Furthermore, the variant absence from public databases reporting genomic variants identified in the healthy population (e.g., gnomAD) allowed us to apply the criteria *Pathogenic Moderate 2* (PM2) [17]. In addition, as previously stated, several in silico prediction tools described the variant as “damaging”, fulfilling the requirements needed to apply the criteria *Pathogenic Supporting 3* (PP3) [17]. Further, the variant is classified as pathogenic in reference databases for variant interpretation such as the HGMD [11] and the Deafness Variation Database [12], which report possibly disease-causing variants identified in patients affected by different conditions. Finally, the variant has already been identified in compound heterozygosis with other variants in two additional USH patients [18,19].

## 4. Discussion

USH1 is an autosomal recessive disease characterized by congenital, bilateral, sensorineural HL, adolescent-onset RP, and vestibular areflexia with variable severity and age onset [3].

An association between USH and various mental and behavioural disorders has been observed, spanning from atypical autism to persecutory delusions, aggressive and anxious behaviour, and schizophrenia [1,9,20,21]. However, despite this evidence, a limited number of studies on psychiatric and psychological issues in this syndrome have been performed [1,4]. In addition, in the literature, there are still conflicting data regarding the prevalence of neuropsychiatric disorders among USH patients, which ranges between ~4 and 23% [1]. These discrepancies may be ascribed, for example, to the impaired communication skills of some patients. This communication barrier may hamper psychometric evaluations, and thus the definition of a mental illness diagnosis [1].

Here, we described a case of a 16-year-old boy with clinical symptoms and a molecular diagnosis of USH1, displaying congenital HL, progressive RP, and behavioural issues.

The proband carries a predicted damaging homozygous variant, c.5985C>A, p.(Tyr1995*), within the *CDH23* (NM_022124.6) gene. *CDH23* is a known USH1 causative gene, and the variant here identified was already reported in the compound heterozygous state in other USH patients [18,19]; however, details regarding clinical, psychological, and behavioural aspects of these patients were not reported. The *CDH23* gene encodes the Cadherin-related family member 23 protein, an atypical cadherin that regulates a variety of cellular processes, including cell–cell adhesion, mechano-electrical transduction, and hair cell bundles’ organisation [22,23]. Notably, this gene is reported to modulate synapse function, neuron growth, and plasticity [24]. Recently, it has been also demonstrated that *CDH23* is expressed in subthalamic and pontine regions in mice models [23], and a similar expression pattern was also identified in the marmoset *Callithrix jacchus*; consequently, a functional conservation of this gene has been hypothesised [23]. Specifically, it was reported that in mice models, *CDH23* regulates the prepulse inhibition (PPI) neural circuit [23], which is a neurological phenomenon known to be altered in several psychiatric disorders, including schizophrenia, Asperger’s syndrome [25], antisocial personality disorder, and violent behaviour [26]. Several hypotheses have been proposed to explain the mechanisms underlying the onset of behavioural and mental disorders in USH syndrome: (i) the stress-related theory, (ii) the brain damage theory; and (iii) the genetic hypothesis [1,21].

The stress-related theory states that the combination of impaired hearing function and progressive decrease in vision acuity has a negative impact on the patient’s psychological well-being, thus resulting in severe psychological stress, depression, communication difficulties, and social isolation [4,21]. Notably, this aspect is worse in USH children, which may mean they encounter more difficulties in language, cognitive development, and social communication, therefore being more susceptible to developing psychiatric and behavioural disorders [1]. In this light, early diagnosis, language rehabilitation, and psychological support are fundamental in preventing these psychiatric issues.

The brain damage theory is derived from the observation of neurological abnormalities in USH patients, such as cerebellar and cerebral atrophy, focal lesions, corpus callosum hypoplasia, fourth ventricle dilatation, and a decrease in intracranial volume [1,27,28], suggesting a pleiotropic effect of USH causative genes.

Finally, a third branch of studies states that many USH genes can predispose individuals to both USH syndrome and mental disorders. Indeed, several associations between USH genes and psychiatric disorders have been reported. As an example, the *CDH23* gene has been linked with schizophrenia [23,29] and bipolar disorders [30]. Specifically, *CDH23* has been associated with a reduced PPI, typically associated with schizophrenia [23,29], and violent behaviour [26]. Further, variants within the *CDH23* gene were detected in patients affected by bipolar disorders [30].

To date, no studies described evidence of a direct genotype–phenotype correlation between USH patients carrying *CDH23* variants and mental/behavioural issues; however, considering the suddenly altered behaviour of the patient and the multiple biological functions of *CDH23*, it can be speculated that this gene could have a pleiotropic effect. Indeed, the concomitant onset of the two phenotypes could be explained by three hypotheses. In detail, (i) the proband’s *CDH23* pathogenic variant may genetically predispose him to mental disorders development; (ii) the altered *CDH23* function may be responsible for an alteration in neurological/brain activity; (iii) the behavioural phenotype could be ascribed to the stress associated with the progressive loss of visual acuity. In this regard, it is possible to hypothesise that the dismal onset of multiple sensory impairments could severely affect the psychological well-being of an individual. Indeed, USH patients are characterised by a double sensory deprivation, which heavily impacts their independence, social interactions, and overall quality of life. Moreover, in the case here described, the onset of the visual impairment was concomitant with the proband’s adolescence, which is a period of life notoriously associated with emotional instability and stress.

However, the combined effect of all three hypotheses cannot be excluded. In particular, even if the emotional and psychological distress associated with the onset of an additional sensory defect (i.e., sight loss) is certainly significant for all patients, in some cases, it could be particularly worsened by a genetic predisposition to the development of mental disorders. This may be specifically relevant for the *CDH23* gene, considering that several literature reports have highlighted its role in brain functionality, and its involvement in several neuropsychiatric conditions. Therefore, there is a growing need to understand the complex interplay between the physiological and genetic factors underlying USH aetiopathogenesis, with the final goal of understanding the heterogeneous landscape of mental and behavioural disorders among USH patients.

In conclusion, this study describes for the first time a possible relevant genotype–phenotype correlation between the *CDH23* gene, USH1, and behavioural problems. However, additional studies will be essential to clarify whether *CDH23* is directly responsible for behavioural issues, or if they should be ascribed to the onset of a dramatic sensorial deprivation. Further, it is possible to speculate that this novel genotype–phenotype correlation could be translated in other USH patients with altered behaviour, hypothesising that other USH genes may also have a similar pleiotropic effect. This consideration highlights the importance of carrying out additional evaluations on this poorly characterized phenotype of USH patients. In fact, the evidence here reported sheds light on the need for continuous clinical monitoring of USH patients to evaluate not only the progression of RP, but also their mental health and brain functionality.

This will allow clinicians to early diagnose any psychiatric and behavioural disorders, in order to prevent the worsening of symptoms with targeted therapeutic strategies, thus improving USH patients’ well-being.

## Figures and Tables

**Figure 1:**
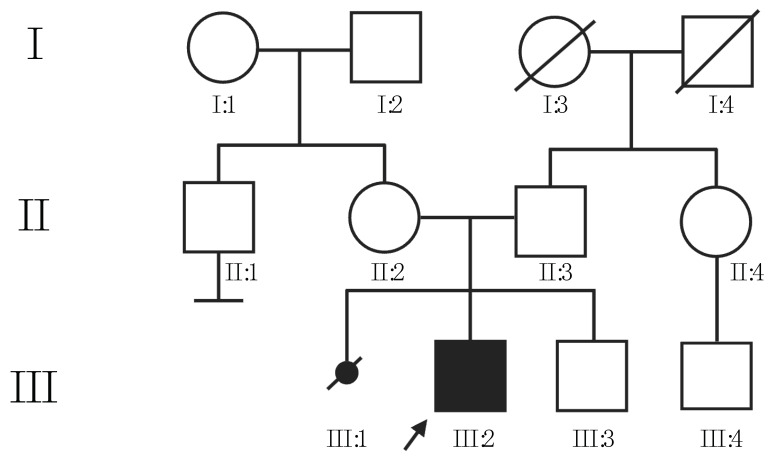
Pedigree of proband’s family. The black arrow indicates the proband. Roman numbers indicate the family’s generations.

## Data Availability

The data presented in this study are available on request from the corresponding author. The data are not publicly available due to privacy restrictions.

## References

[1] Domanico D., Fragiotta S., Cutini A., Grenga P.L., Vingolo E.M. (2015). Psychosis, Mood and Behavioral Disorders in Usher Syndrome: Review of the Literature. Med. Hypothesis Discov. Innov. Ophthalmol..

[2] Delmaghani S., El-Amraoui A. (2022). The Genetic and Phenotypic Landscapes of Usher Syndrome: From Disease Mechanisms to a New Classification. Hum. Genet..

[3] Koenekoop R.K., Arriaga M.A., Trzupek K.M., Lentz J.J. (2020). Usher Syndrome Type I. GeneReviews.

[4] Domanico D., Fragiotta S., Trabucco P., Nebbioso M., Vingolo E.M. (2012). Genetic Analysis for Two Italian Siblings with Usher Syndrome and Schizophrenia. Case Rep. Ophthalmol. Med..

[5] Arcous M., Putois O., Dalle-Nazébi S., Kerbourch S., Cariou A., Ben Aissa I., Marlin S., Potier R. (2020). Psychosocial Determinants Associated with Quality of Life in People with Usher Syndrome. A Scoping Review. Disabil. Rehabil..

[6] Dean G., Orford A., Staines R., McGee A., Smith K.J. (2017). Psychosocial Well-Being and Health-Related Quality of Life in a UK Population with Usher Syndrome. BMJ Open.

[7] Roborel de Climens A., Tugaut B., Piscopo A., Arnould B., Buggage R., Brun-Strang C. (2020). Living with Type I Usher Syndrome: Insights from Patients and Their Parents. Ophthalmic Genet..

[8] Waldeck T., Wyszynski B., Medalia A. (2001). The Relationship between Usher’s Syndrome and Psychosis with Capgras Syndrome. Psychiatry.

[9] Hess-Röver J., Crichton J., Byrne K., Holland A.J. (1999). Case Report: Diagnosis and Treatment of a Severe Psychotic Illness in a Man with Dual Severe Sensory Impairments Caused by the Presence of Usher Syndrome. J. Intellect. Disabil. Res..

[10] Rijavec N., Grubic V.N. (2009). Usher Syndrome and Psychiatric Symptoms: A Challenge in Psychiatric Management. Psychiatr. Danub..

[11] Stenson P.D., Mort M., Ball E.V., Chapman M., Evans K., Azevedo L., Hayden M., Heywood S., Millar D.S., Phillips A.D. (2020). The Human Gene Mutation Database (HGMD®): Optimizing Its Use in a Clinical Diagnostic or Research Setting. Hum. Genet..

[12] Azaiez H., Booth K.T., Ephraim S.S., Crone B., Black-Ziegelbein E.A., Marini R.J., Shearer A.E., Sloan-Heggen C.M., Kolbe D., Casavant T. (2018). Genomic Landscape and Mutational Signatures of Deafness-Associated Genes. Am. J. Hum. Genet..

[13] Adzhubei I., Jordan D.M., Sunyaev S.R. (2013). Predicting Functional Effect of Human Missense Mutations Using PolyPhen-2. Curr. Protoc. Hum. Genet..

[14] Ng P.C., Henikoff S. (2003). SIFT: Predicting Amino Acid Changes That Affect Protein Function. Nucleic Acids Res..

[15] Limongelli I., Marini S., Bellazzi R. (2015). PaPI: Pseudo Amino Acid Composition to Score Human Protein-Coding Variants. BMC Bioinform..

[16] Quang D., Chen Y., Xie X. (2015). DANN: A Deep Learning Approach for Annotating the Pathogenicity of Genetic Variants. Bioinformatics.

[17] Richards S., Aziz N., Bale S., Bick D., Das S., Gastier-Foster J., Grody W.W., Hegde M., Lyon E., Spector E. (2015). Standards and Guidelines for the Interpretation of Sequence Variants: A Joint Consensus Recommendation of the American College of Medical Genetics and Genomics and the Association for Molecular Pathology. Genet. Med..

[18] Ma J., Ma X., Lin K., Huang R., Bi X., Ming C., Li L., Li X., Li G., Zhao L. (2023). Genetic Screening of a Chinese Cohort of Children with Hearing Loss Using a Next-Generation Sequencing Panel. Hum. Genom..

[19] Vaché C., Besnard T., Blanchet C., Baux D., Larrieu L., Faugère V., Mondain M., Hamel C., Malcolm S., Claustres M. (2010). Nasal Epithelial Cells Are a Reliable Source to Study Splicing Variants in Usher Syndrome. Hum. Mutat..

[20] Viala A., Nicot T., Levy F., Vacheron M.N. (2009). A Case of Usher’s Syndrome Associated with Psychotic Symptoms: Diagnosis and Follow-up in a Psychiatric Unit. Encephale.

[21] Dammeyer J. (2012). Children with Usher Syndrome: Mental and Behavioral Disorders. Behav. Brain Funct..

[22] Müller U. (2008). Cadherins and Mechanotransduction by Hair Cells. Curr. Opin. Cell Biol..

[23] Balan S., Ohnishi T., Watanabe A., Ohba H., Iwayama Y., Toyoshima M., Hara T., Hisano Y., Miyasaka Y., Toyota T. (2021). Role of an Atypical Cadherin Gene, Cdh23 in Prepulse Inhibition, and Implication of CDH23 in Schizophrenia. Schizophr. Bull..

[24] Terracciano A., Sanna S., Uda M., Deiana B., Usala G., Busonero F., Maschio A., Scally M., Patriciu N., Chen W.M. (2008). Genome-Wide Association Scan for Five Major Dimensions of Personality. Mol. Psychiatry.

[25] Swerdlow N.R., Braff D.L., Geyer M.A. (2016). Sensorimotor Gating of the Startle Reflex: What We Said 25 Years Ago, What Has Happened since Then, and What Comes Next. J. Psychopharmacol..

[26] Kumari V., Das M., Hodgins S., Zachariah E., Barkataki I., Howlett M., Sharma T. (2005). Association between Violent Behaviour and Impaired Prepulse Inhibition of the Startle Response in Antisocial Personality Disorder and Schizophrenia. Behav. Brain Res..

[27] Koizumi J., Ofuku K., Sakuma K., Shiraishi H., Saakio M., Nawano S. (1988). CNS Changes in Usher’s Syndrome with Mental Disorder: CT, MRI and PET Findings. J. Neurol. Neurosurg. Psychiatry.

[28] Tamayo M.L., Maldonado C., Plaza S.L., Alvira G.M., Tamayo G.E., Zambrano M., Frias J.L., Bernai J.E. (1996). Neuroradiology and Clinical Aspects of Usher Syndrome. Clin. Genet..

[29] Greenwood T.A., Lazzeroni L.C., Maihofer A.X., Swerdlow N.R., Calkins M.E., Freedman R., Green M.F., Light G.A., Nievergelt C.M., Nuechterlein K.H. (2019). Genome-Wide Association of Endophenotypes for Schizophrenia From the Consortium on the Genetics of Schizophrenia (COGS) Study. JAMA Psychiatry.

[30] Han M.R., Han K.M., Kim A., Kang W., Kang Y., Kang J., Won E., Tae W.S., Cho Y., Ham B.J. (2019). Whole-Exome Sequencing Identifies Variants Associated with Structural MRI Markers in Patients with Bipolar Disorders. J. Affect. Disord..

